# The effects of social participation on social integration

**DOI:** 10.3389/fpsyg.2022.919592

**Published:** 2022-09-02

**Authors:** Peng Xie, Qinwei Cao, Xue Li, Yurong Yang, Lianchao Yu

**Affiliations:** ^1^School of Economics and Management, Wuhan University, Wuhan, China; ^2^School of Management, Wuhan University of Technology, Wuhan, China; ^3^School of Foreign Studies, Zhongnan University of Economics and Law, East Lake High-tech Development Zone, Wuhan, Hubei, China; ^4^School of Business, Chongqing City Vocational College, Chongqing, China; ^5^School of Management, Lanzhou University, Lanzhou, China

**Keywords:** *hukou*, social integration, formal social participation, informal social participation, migrants

## Abstract

With the fast expansion of urbanization, temporary migrants have become a large demographic in Chinese cities. Therefore, in order to enhance the social integration of the migrant population, scholars and policymakers have an urgency to investigate the influencing factors of the integration progress. Prior studies regarding social integration have neglected to examine this topic from the perspective of social participation. Empirical research is conducted based on the data of 15,997 migrants across eight cities in the 2014 wave of National Migrant Population Dynamic Monitoring Survey (MDMS) in China. Hierarchical linear models were used to test the hypotheses regarding the impacts of formal social participation (FSP) and informal social participation (ISP) on social integration. Community type, neighbor composition, hometown pressure, withdrawal guarantee, and constraints of *hukou* were examined as moderators. FSP and ISP possess different features such as operating with distinct modes, providing different services. Members within the organizations also entail different rights and responsibilities, providing them with different types of social capital and psychological perceptions. Hence, this study strived to identify the effects of social participation behaviors on social integration from a social capital perspective. The results revealed that social participation is positively linked to social integration. We also distinguished between FSP and ISP of migrants to investigate the boundary effects of different types of social participation on social integration. The findings provide both theoretical and practical implications for scholars as well as policymakers on issues regarding the social integration of migrants.

## Introduction

The National Health Commission of the People’s Republic of China (NHCPPC) released the 2018 Report on China’s Migrant Population Development. The report exhibited that the scale of China’s migrant population has reached 244.5 million, approximately accounting for 17.5% of the total population of China (NHCPPC, 2018). This massive expansion of the migrant population is positively correlated to the accelerating urbanization process of China as this demographic has become a critical driving force of the country’s economy by providing abundant labor and human resources (Lin et al., 2020; Xia and Ma, 2020). In order to achieve steady and harmonious development of the society, the Chinese government has attached significant attention to the social integration of the migrant population. However, due to the inertia of the urban-rural dual system that affects the migrants’ rights to social welfare and factors that generate psychological barriers, migrants are facing difficulties integrating into the local society (Lu et al., 2013; Gu and Yeung, 2020; Lin et al., 2020).

Social integration of the migrant population can promote economic development as well as maintain social harmony. Hence, the driving factors of migrants’ social integration have attracted widespread attention of scholars and have become a major topic in sociology (Wang and Fan, 2012; Chen and Wang, 2015; Body-Gendrot and Martiniello, 2016; Yang et al., 2020). Established on the basis of social capital theory, our research aims to focus on the drivers of migrants’ identity. Specifically, we investigate the impact of formal social participation (FSP) and informal social participation (ISP) on social integration from the perspective of psychological identity. As many scholars emphasized the landmark role of the psychological identity, this study focuses on the factors influencing migrants’ identity. Based on social capital theory, we aim to examine the effect of FSP and ISP on social integration from the perspective of psychological identity as well as the moderators that affect this relationship.

This research contributes to the literature on social capital theory and social integration in the following ways. First, our study extended the theory of social capital by examining social participation from the viewpoints of FSP and ISP. Amongst the related studies of social capital theory, the measurement of social capital is an important focus of research. Different studies on the measurement of social capital vary depending on different research contexts. Second, this research explored the effects of social participation on migrants’ psychological identity, which contributes to a better understanding of the relationship between social participation and social integration. Prior research on social integration literature focused more on the objective aspects of economic, political, and cultural integration while neglecting the subjective aspects of integration such as psychological integration. Third, this research unveiled important moderating effects. The findings not only help to enrich the literature on social integration but also extend the research on social capital theory. From a practical perspective, the results of our research can make policymakers more aware of the influences of organizational activities on migrants’ social identity and self-perceptions. Further, this research also provides important implications for the self-development and mental health of the migrant population.

The remaining section of this paper is organized as follows. First, we gave a review of the prior research and literature on social integration, followed by a set of developed research hypotheses. Then, we explained the research hypotheses by survey data. In the “Data and methods” Section, we elaborated the design for this research, including data, variables and measurement, and model specification. Finally, we presented the empirical findings and concluded with a discussion as well as limitations and orientations for future research.

## Conceptual framework and hypothesis

### Social integration

Social integration is regarded as a multidimensional concept, which describes the capacity of people to participate in social, cultural, economic, and political life in the local community (Brydsten et al., 2019). Although there are differences in the definition and measurement of social integration depending on the context. Most scholars measure social integration from the aspects of economic integration, political integration, cultural integration, and psychological integration. Studies found that economic integration is considered as the starting point of social integration, which plays a fundamental role; While psychological integration, established on the dimensions of economic and political integration, is considered as the advanced and final stage of social integration (Lu et al., 2013; Gu and Yeung, 2020; Lin et al., 2020).

As an advanced stage of social integration, psychological integration is a critical link in the study of immigration integration. From the perspective of diversity, psychological integration can be understood as a situation in which social unity exists among different cultural or racial groups (Adachi, 2011). People with different backgrounds share common norms and rules of society and feel a sense of belonging to the host place and society, although they may have different habits, interests, or religions (Adachi, 2011). Subjective sense of belonging and actual rights and responsibilities as actual citizens are the two important aspects of social integration (Xia and Ma, 2020), which are understood as the migrant’s physical and psychological connections with the host members, such as their neighbors in the community. When migrants share a strong sense of identity and belonging to the host city, they regard themselves as a member of the community. In other words, when migrants fully achieve social integration, they will care about the development and growth of the host city, and their values and behaviors will be enhanced according to the city (Cinnirella, 1996; Adachi, 2011; Fernando and Patriotta, 2020). Based on previous studies, this article focuses on social integration at the psychological level, which is considered the ultimate stage of social integration.

In Western nations, there is a long history of research into the social integration of migrants both at the domestic and international levels. Unlike the mobility of migrants in developed countries where migration mostly takes place on a transnational level, the migration of China and other developing countries usually occurs in-between cities (Chen and Wang, 2015; Brydsten et al., 2019; Lin et al., 2020). However, a majority of the migrant population all encounter similar integration obstacles and challenges in terms of cultural, economic, and social differences compared with the local residents (Chen and Wang, 2015; Wang et al., 2016). Our research aims to investigate how Chinese domestic migrants can better socially integrate into the host city. This study not only makes up for the lack of domestic research but also provide important guidance value for emerging entities on formulating pertinent policies and promoting high-quality urbanization. In view of this, this paper attempts to examine the important factors that influence psychological integration from the perspective of social capital, and explore the boundary mechanism of this underlying relationship.

### Hypotheses

#### Social participation and social integration

Social organizations provide the opportunity for migrants to perceive and detect the differences between themselves and the locals. Becoming aware and accustomed to the cultural diversity in the host place is an important process for achieving social integration (Adachi, 2011). For this reason, participation in organizations in the host city provide the opportunity for migrants to be exposed to cultural diversity and allowing themselves to adjust to the host culture accordingly (Fisher, 2007; Zou and Deng, 2020). Further, cultural diversity between regions in China is not substantial, and is often recognized and accepted along with more interactions when participating in social organizations (Yue et al., 2013).

Social organizations are a platform for migrants to obtain useful social resources, especially for rural migrants who face many disadvantages and prejudice brought by the “urban-rural distinctions.” This prejudice often places them on the passive side of social integration. In order to change their passive status in the local community, it is necessary for the migrants to adapt to the host environment and then expand their social resources. For obtaining development resources and establishing social ties, individuals actively participate in organizational activities and attempt to cultivate contacts with other people (Huang et al., 2018). Depending on the different types of organizational activities, our research defines general organizational activities of the migrant population as informal social participation and formal organizational activities as formal social participation. Formal organization, which refers to the outcome of goal specification and structure formalization, is a common, rational system that transcends any independent individual. On the contrary, informal organization is an organizational structure that lacks institutionalization, structure, and formalization, such as villager gatherings or alumni associations. Especially, the migrants can benefit from participating in the local organizations, because formal and informal organizations are the combination of different social networks which contains various information, capital, and technical resources (Davis and Newstrom, 1981; Sharicz et al., 2010). In addition, the Chinese have a deep-rooted idea that one relies on their parents at home and on friends outside (Huang et al., 2018). Therefore, for migrants with weak resource endowment, they can accumulate resources and social capital and improve collective action capabilities through participating in different organizations (Yue et al., 2013).

Group unity and group commonality can enhance the migrant population’s sense of belonging and social integration. Generally, members in organizations share the same goals and values, which can ultimately enhance the overall cohesion of the group. In addition, through providing a low-cost multi-channel for migrants to deal with various conflicts and disputes, organizations can facilitate the gears of relationship between migrants and locals. Therefore, the advantages of coordination mechanism, trust mechanism, and reciprocal norms of organizations enable migrants to develop a sense of belonging and approbation to the host cities. Based on the above, we put forward the following hypotheses:

H1a: Informal social participation of migrants will positively influence social integration.

H1b: Formal social participation of migrants will positively influence social integration.

#### The moderating effect of the environment of community and neighbor composition

The environment of the community plays a crucial role in the social integration of migrants. Misunderstandings and incompatibility between migrants and their neighbors may lead to higher psychological distance and low motivation for social integration (Li et al., 2012; Wang and Ning, 2016).

For that reason, strengthening social integration always starts with their initial settlements, that is, the communities (Lin et al., 2020). The environment of community, including the physical hardware facilities and cultural norms which influence migrant psychological status and perceptions. Specifically, cultural norms are a reflection of the local members’ common attitude and convictions. Generally, better community signifies better facilities and neighbors who always want to maintain a harmonious relationship with one another and have an open perspective (Zou and Deng, 2020). On one hand, the harmony and friendship in better communities can increase communication and enhance understanding between migrants and local neighbors thus improving the migrants’ experience of interaction with the locals and enhance their sense of fairness and belonging. Furthermore, neighbors with inclusive and open perspectives are able to provide better emotional and social support. This mutual understanding and support can serve as a catalyst for increasing migrants’ confidence in social interactions with the locals (Li et al., 2012; Zou and Deng, 2020). Therefore, migrants in better communities will adhere to the common goals and norms, thus improving the positive effect of organization on migrants’ identification.

Moreover, neighbor composition also play a major role in the social integration of migrants as frequent interactions between locals and migrants generate complex effects (Li et al., 2012). Without suitable ways, the interaction may deepen contradictions and confusions between migrants and locals. However, based on interests and hobbies, they are able to share the dynamic and active atmosphere of informal organization, which help migrants keep harmonious relationships with native neighbors. Hence, informal organization help migrants who live in a community with more natives to reduce their misunderstandings and increase their social integration.

The informal interaction between migrants and local residents could build carefree atmosphere in an easy and enjoyable way. While on the contrary, formal organizations emphasize efficiency and regulation. This type of gathering neglects the emotional needs of the migrants, therefore more likely to cause psychological conflicts and negatively affect their social integration. The possibility of conflicts between migrants and local residents are likely to increase with higher percentage of local residents in the neighborhood, which may strengthen the sense of unwelcomeness and the perception of unfair hierarchy. Hence, individuals are more sensitive to formal organizational hierarchy and power distance, which reduce their social identification. Taken together, community type and neighbor composition could be potential moderators between social integration and ISP for migrants. In light of this, we propose four sub-hypotheses:

H2a: Community type will positively moderate the positively impact of ISP on social integration.

H2b: Community type will positively moderate the positively impact of FSP on social integration.

H2c: Neighbor composition will positively moderate the positively impact of ISP on social integration.

H2d: Neighbor composition will negatively moderate the positively impact of FSP on social integration.

#### The moderating effect of hometown pressure

According to existing literature, there is a negative effect of hometown pressure on social integration. Hometown pressure and distractions could divert their attention from life and work in local cities. Subject to mood swings and emotional stress, migrants are more likely to experience negative sentiments, such as fear, sadness, and anxiety. Although informal organizations have a positive effect on relieving work and life pressure, the emotional communication in informal organizations make it easier to stimulate the passive emotions of missing hometown, given that hometown affairs are the main content and topics of exchanges between migrants and locals. Therefore, the worries about their hometown are more significant in the emotional communication in informal organizations, thus impeding migrants’ motivation to integrate into the city. We present the following research hypothesis:

H3: Hometown pressure will negatively moderate the positive impact of ISP on social integration.

#### The moderating effect of perceived social status

Local residents often hold prejudice and discrimination for migrants, especially migrant workers. They are convinced that the presence of migrant workers negatively influences the local culture and employment atmosphere as well as causes various social problems (Fisher, 2007). However, with the rise of migrants’ social status, their perception of social exclusion and relatively disadvantaged position become relatively weak (Yoon et al., 2012). Specifically, higher perceived social status prevents migrants from interpreting the contradictions and disputes as exclusion and discrimination, thus avoiding the information processing model associated with the negative awareness of locals’ reactions. Further, with the increased fulfillment and confidence in their own social status, migrants are more active in engaging in communication and interaction with others. Therefore, migrants with positive perceived social status are more confident and more powerful in formal organizations, where the members’ efficiency to achieve the organization’s purpose is an important evaluation standard and the hierarchical relationship between members is one of the prominent features. In light of this, we proposed our fourth hypothesis as follows:

H4: Perceived social status will positively moderate the positive impact of FSP on social integration.

#### The moderating effect of withdrawal guarantee

Farmers account for the majority of China’s migrant population whose main sources of income before immigration comes from agriculture. Particularly, the farmers’ income heavily depends on the area of their land. At the same time, they attach great importance to the right to use cultivated land and consider land as a major part of family asset.

Owning land provides an important psychological foundation for them to participate in activities and organizations. With the land in their hometown as a guarantee, the migrant does not have to worry too much about the results of participating in organizations. Acting as a reassurance and guarantee, land provides the confidence for migrants during interactions with locals in different organizations. As psychological burden reduces and positive intentions increases, migrants are more likely to participate in organizational activities with an optimistic attitude. In light of this, we propose two sub-hypotheses as follows:

H5a: Withdrawal guarantee will positively moderate the impact of ISP on social integration.

H5b: Withdrawal guarantee will positively moderate the impact of FSP on social integration.

**FIGURE 1:**
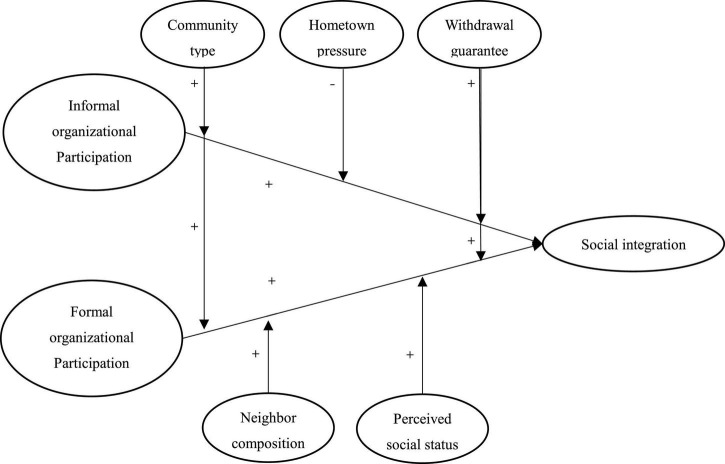
Conceptual framework.

In summary, we constructs the theoretical framework, as shown in Figure 1.

## Data and methods

### Survey and dataset

The data for this study was collected from the 2014 wave of National Migrant Population Dynamic Monitoring Survey (MDMS) in China. After eliminating two respondents who didn’t answer the relative questions, we got a final sample with 15,997 migrants.^1^ We chose the MDMS dataset as it served our research goals. First, the dataset of the 2014 MDMS contained the focal items in our research questions–social participation, social integration, perceived social status, hometown pressure, withdrawal guarantee, and community environment. Second, the extensive information of this dataset helped us to rule out the covariates of social integration suggested by the literature, thus helping us to reduce the potential confounding effects. Third, reasonable sampling method ensures the representativeness of the samples. In addition, the eight cities (i.e., Beijing, Chengdu, Jiaxing, Qingdao, Xiamen, Shenzhen, Zhengzhou, and Zhongshan), chosen by following the guidelines of the National Population and Family Planning Commission of China, are distributed in different regions of China, and all have large-scale floating population. Lastly, the 2014 MDMS data is of high quality and accessibility.

### Variables and measurement

We used social identity to capture social integration. According to the answers of the question whether they regard themselves as the local, we coded the answer “Yes” as 1 and “No” as 0. Hence, we used social identity in our main test and presented our logistic regression models, with social identity as a dummy.

We categorized social participation into two types: FSP and ISP. Regarding organizational involvement, respondents were asked a question: “Are you currently a member of the following organizations?” To examine the impact of organization involvement, we classified the multiple options of this question into formal and informal organizations. Specifically, the formal group contains “labor union,” “mobile party branch,” and “local party branch,” while the informal group includes “volunteer association,” “student association,” “chamber of commerce,” and “fellow club.” According to the answers of the respondents, the FSP was coded “1” if answers include any formal organizations and “0” otherwise. Similarly, the ISP was coded “1” if respondents chose any informal organizations otherwise “0.”

To understand the constraints and disadvantages caused by the *hukou* system, we counted the number of five kinds of insurance (i.e., unemployment insurance, urban employee pension insurance, endowment insurance for urban residents, a housing provident fund, and a new agricultural insurance) to measure the constraints of *hukou*. *Hukou* system has great impact on the social welfare of migrants from foreign regions (Chan, 2009; Huang et al., 2014). Moreover, the degree of relative insurance participation is a real reflection of the function of the social security system for migrants. Therefore, we think using the number of insurance that individuals did not participating in can reflect the constrains of the *hukou* system. Specifically, bigger number of insurance signifies the more barriers the respondent is facing.

To account for the relative perceived social status of the respondents, we used their responses to the four items for the question: (1) “Compared with your relatives, friends and colleagues, where are you in the ten-level social status?” (2) “Compared with people in the whole society, where are you in the ten-level social status?” (3) “Compared with your relatives, friends and colleagues in your current place of residence, what is the degree of respect you experience in the ten levels?” (4) “Compared with people in the whole society, what is the degree of respect you experience in the ten levels” Using factor analysis, we extracted a single factor score to indicate the perceived social status. Another individual perception of respondents we included in this study was the perceived social exclusion. We used three items in questionnaires for the perceived social exclusion of household population: (1) “I think the locals are willing to accept me as one of them.” (2) “I feel that the locals do not want to be my neighbors.” (3) “I feel that the locals do not like me.” (4) “I feel that the locals look down on me.” (“extremely disagree” 1 to 4 “extremely agree”). Specifically, after the first item was reverse coded, the scores of items were combined into a single score, respectively using factor analysis.

We included the income to capture the individual’s labor market status (Chen and Liu, 2016). This was measured by the natural logarithm of average monthly total household income in the local area. In addition, we included the natural logarithm of average monthly total household expenditure to capture family’s financial status. Moreover, we captured the hometown pressure by counting how many events the migrant worry about (i.e., raising children, looking after the elderly, the loneness of spouse, children’s education expenses, labor shortage for family working, lack of money for sick family members, farming and the other for respondents specifying themselves). Otherwise, according to the question “How many acres of land does your family have in the place of residence (hometown)?” we used the natural logarithm of the cultivated area to capture the withdrawal guarantee for migrants.

We captured the effect of the living environment on the social integration by community type and neighbor composition (Li et al., 2012; Zou and Deng, 2020). The community type was a dummy variable, with the communities having a good living condition as 1 (i.e., villa area or commercial housing community, affordable housing community, institutions and institutions community, and industrial and mining enterprise community) and the other communities as 0 (i.e., unrenovated old town, suburban junction, rural community, and others). In the questionnaire, all respondents were asked whom their neighbors are. They were given the following possible options: “local citizens,” “foreigners,” and “similar number of foreigners and locals.” We recoded this item into a continuous variable, with foreigners = 1, similar number of foreigners and locals = 2, and local citizens = 3. Therefore, the neighbor composition reflects the scale of the target group of integration in their neighborhood.

Following previous literature, we also controlled some demographic variables and other relative variables in the models (Rublee and Shaw, 1991; De Paola and Brunello, 2016; Huang et al., 2018). Specifically, at the individual level, the models included the sex (male = 1, female = 0), age, types of *hukou* (rural = 1, others = 0), ethnic and racial identity (Han = 1, minorities = 0), indicators for different marital status (i.e., unmarried, newly married, and remarried), occupation indicators (e.g., professional technicians), educational attainment, employment status (employee = 1, others = 0), number of community activities (e.g., community sports activities, social welfare activities, and owner committee activities) the individual participated in, employment period of labor contract the respondent signed (long-term contract = 1), labor contract status (no labor contract = 1), free government-provided training (received = 1, not received = 0), and working pressure, as the average hours of work per day in the last month (or last employment). In addition, especially for the situation about migration (Wang and Fan, 2012; Huang et al., 2018), we also included the mobility reason (for business = 1, others = 0), the duration of current mobility and ranges of migration (interprovincial and intra-provincial mobility), and the participation of free government-provided training (participated = 1, not participated = 0).

At the city level, we controlled cities’ natural logarithm of gross domestic product (GDP) of focal city in 2013, and proportion of floating population to total population of the relative province according to the Sixth national Population Census of China, due to the floating population data was unavailable in city level. Descriptive statistics of all variables were reported in Table 1.

**TABLE 1:** Descriptive statistics of key variables.

Variables	Migrants	
	
	Frequency	Percentage or mean (SD in parentheses)
Local identity	3,516	22.0%
Willingness to integration		0.0 (3.3)
Informal organization participation	3,389	21.2%
Formal organization participation	1,535	9.6%
Male (Female = 0)	8,798	55.0%
Age		32.7 (8.7)
Educational attainment		3.5 (1.0)
Ethnic minority (Han = 1)	15,434	96.5%
**Marriage status**		
Unmarried	4,056	25.4%
Newly married	11,538	72.1%
Remarried	169	1.1%
Employee	10,098	63.1%
Employer	1,083	6.8%
*Hukou* (rural = 1)	13,757	86.0%
Constraints of *hukou*		3.9 (1.0)
Working pressure		8.8 (2.8)
Perceived social exclusion		0.0 (2.0)
Perceived social status		0.0 (1.8)
**Labor contract**		
Long-term labor contract (long = 1)	7,064	44.2%
Labor contract (1 = no labor contract)	5,895	36.9%
Household expenditure		7.8 (0.6)
Household income		8.6 (0.6)
Community type (well condition = 1)	6,612	41.3%
Neighbor composition		1.8 (0.8)
Community activities		0.7 (1.0)
Government-provided training	4,742	29.6%
Mobility reason (for business = 1)	15,188	94.9%
**Ranges of migration**		
Interprovincial	8,769	54.8%
Intra-provincial	6,635	41.5%
Hometown pressure		1.7 (1.5)
Withdrawal guarantee		1.2 (0.8)
Duration of current mobility		5.3 (4.4)
GDP		7.8 (0.3)
Proportion of floating population		0.3 (0.2)

### Model specification

Because the respondents nested within cities, the basic data structure is hierarchical. Based on the proposed conceptual framework, multilevel logistic model (MLM) was used to estimate the effects of social participation on migrants’ social identity (Goldstein, 2011). Specifically, our hierarchical models treated the respondents as Level 1 (the individual level) and the eight cities as Level 2 (the city level). The functional forms of the hierarchical linear model (HLM) are as follows:


(1) $$\log\left(\frac{S\,I_{i\,c}}{1-S\,I_{i\,c}}\right)=\propto_{0\,c}+\sum \alpha_{i\,c}X_{i\,c}+\varepsilon_{i\,c}$$

where *SI*_*ic*_ denotes the social identity probability for individual i in city c, *X*_*ic*_ denotes a list of individual-level variables, and ε_*i*_ captures the random errors operating at the individual level.


(2) $$\propto_{0\,c}=\gamma_{00}+\sum \gamma_{0\,c}Z_{i\,c}+\mu_{0\,c}$$

(3) $$\propto_{i\,c}=\gamma_{i\,0}$$

where θ_0*c*_ denotes a list of city-level variables, and μ_0*c*_ captures the random errors operating at the city level.

The establishment of a multi-level model is mainly to study the impact of social participation on the social identity. For the sake of model minimalism, the random effect of variables at the individual level was ignored. In addition, according to the literature, there is no reason to assume that the characteristics of the city level have the structural effect on individual’s community environment, neighbor composition. Overall, we used the random effects model to examined our hypothesis, specifically, the intercept varies around different cites’ mean intercept, and the slopes are fixed.

At the individual level, all model control for individual demographic variables: gender, age, racial identity, the types of *hukou*, marital status, occupation, educational attainment, employment status, community activities, and employment period of labor contract; family characteristic variable: income and expenditure; community environment: community type and neighbor composition; and other variables: perceived social status, perceived social exclusion, the constraints of *hukou*, and working pressure. Especially, we also controlled the mobility reason, the duration of current mobility, ranges of their mobility, their free government-provided training, hometown pressure and withdrawal guarantee for the migrants. At the city level, we included the GDP and floating population ratio.

## Results

The main goals of the empirical analysis were to quantitatively assess the effect of social participation on individual’s social identity, and the interaction effects of social participation and other important antecedent variables on the social identity. Table 1 provided an overview of variables. Table 2 presented a series of models on FSP and ISP on social identity. Some boundary effects of social participation on social integration was confirmed by regression analyses in which the willingness to integration was used as dependent variable.^2^

**TABLE 2:** Predictors of migrants’ local identity by logistic models and GHLM.

	Logistic			Generalized hierarchical linear model		
		
	(1)	(2)	(3)	(4)	(5)	(6)
Informal social participation (ISP)	0.119* (0.051)	0.098^†^ (0.051)	−0.011 (0.075)	0.024** (0.008)	0.020* (0.008)	0.004 (0.011)
Formal social participation (FSP)	0.167* (0.071)	0.162* (0.071)	−0.051 (0.102)	0.031** (0.012)	0.031** (0.012)	−0.004 (0.016)
Gender	0.024 (0.042)	0.016 (0.042)	0.019 (0.042)	−0.0001 (0.006)	−0.001 (0.006)	−0.001 (0.006)
Age	0.005^†^ (0.003)	0.006^†^ (0.003)	0.005^†^ (0.003)	0.0004 (0.0005)	0.0004 (0.0005)	0.0004 (0.0005)
Ethnic minority	0.023 (0.122)	0.022 (0.122)	0.020 (0.122)	0.002 (0.017)	0.002 (0.017)	0.003 (0.017)
Educational attainment	0.051* (0.025)	0.048^†^ (0.025)	0.047^†^ (0.025)	0.011** (0.004)	0.011** (0.004)	0.011** (0.004)
Unmarried	−0.250 (0.167)	−0.263 (0.168)	−0.265 (0.168)	−0.011 (0.028)	−0.015 (0.028)	−0.014 (0.028)
Newly married	−0.079 (0.157)	−0.104 (0.158)	−0.102 (0.159)	−0.001 (0.026)	−0.005 (0.026)	−0.005 (0.026)
Remarried	−0.034 (0.240)	−0.037 (0.241)	−0.035 (0.242)	0.008 (0.040)	0.007 (0.040)	0.009 (0.040)
*Hukou*	−0.341*** (0.063)	−0.347*** (0.063)	−0.354*** (0.063)	−0.059*** (0.011)	−0.059*** (0.011)	−0.060*** (0.011)
Constraints of *hukou*	−0.088*** (0.026)	−0.089*** (0.026)	−0.088*** (0.026)	−0.016*** (0.004)	−0.016*** (0.004)	−0.016*** (0.004)
Employee	0.943 (1.299)	0.977 (1.264)	0.981 (1.264)	0.098 (0.160)	0.114 (0.160)	0.116 (0.160)
Long-term labor contract	−0.021 (0.063)	−0.032 (0.063)	−0.023 (0.064)	−0.007 (0.009)	−0.009 (0.009)	−0.008 (0.009)
Labor contract	0.854 (1.299)	0.863 (1.264)	0.874 (1.264)	0.091 (0.160)	0.103 (0.160)	0.107 (0.160)
Working pressure	−0.030** (0.010)	−0.028** (0.010)	−0.028** (0.010)	−0.004* (0.002)	−0.003* (0.002)	−0.003* (0.002)
Perceived social exclusion	−0.172*** (0.011)	−0.159*** (0.011)	−0.160*** (0.011)	−0.026*** (0.002)	−0.024*** (0.002)	−0.024*** (0.002)
Household expenditure	0.088^†^ (0.049)	0.086^†^ (0.049)	0.083^†^ (0.050)	0.021** (0.008)	0.021** (0.008)	0.020** (0.008)
Household income	0.008 (0.051)	−0.045 (0.051)	−0.048 (0.051)	0.003 (0.008)	−0.005 (0.008)	−0.005 (0.008)
Community type	0.279*** (0.044)	0.288*** (0.044)	0.199*** (0.051)	0.047*** (0.007)	0.049*** (0.007)	0.034*** (0.008)
Neighbor composition	0.341*** (0.027)	0.331*** (0.027)	0.324*** (0.031)	0.045*** (0.004)	0.043*** (0.004)	0.040*** (0.005)
Community activities	0.084*** (0.021)	0.078*** (0.021)	0.082*** (0.021)	0.020*** (0.004)	0.019*** (0.004)	0.020*** (0.004)
Duration of current mobility	0.022*** (0.005)	0.022*** (0.005)	0.023*** (0.005)	0.004*** (0.001)	0.004*** (0.001)	0.004*** (0.001)
Interprovincial	−0.814*** (0.095)	−0.816*** (0.095)	−0.818*** (0.096)	−0.158*** (0.018)	−0.157*** (0.018)	−0.155*** (0.018)
Intra-provincial	−0.509*** (0.092)	−0.506*** (0.093)	−0.505*** (0.093)	−0.101*** (0.017)	−0.100*** (0.017)	−0.100*** (0.017)
Mobility reason	−0.068 (0.102)	−0.081 (0.102)	−0.080 (0.102)	−0.020 (0.016)	−0.022 (0.016)	−0.022 (0.016)
Hometown pressure	−0.077*** (0.015)	−0.069*** (0.015)	−0.049** (0.017)	−0.011*** (0.002)	−0.010*** (0.002)	−0.006* (0.003)
Withdrawal guarantee	−0.072* (0.028)	−0.072* (0.028)	−0.085** (0.032)	−0.006 (0.004)	−0.006 (0.004)	−0.008 (0.005)
Government-provided training	0.073 (0.046)	0.065 (0.046)	0.065 (0.046)	−0.009 (0.008)	−0.011 (0.008)	−0.010 (0.008)
GDP				0.033 (0.079)	0.037 (0.079)	0.037 (0.078)
Proportion of floating population				−0.039 (0.133)	−0.042 (0.132)	−0.041 (0.132)
Perceived social status		0.104*** (0.012)	0.106*** (0.014)		0.016*** (0.002)	0.016*** (0.002)
ISP × Community type			0.186^†^ (0.100)			0.037* (0.016)
ISP × Neighbor composition			0.131* (0.063)			0.029** (0.010)
ISP × Hometown pressure			−0.074* (0.035)			−0.014** (0.005)
ISP × Perceived social status			−0.041 (0.026)			−0.005 (0.004)
ISP × Withdrawal guarantee			0.090 (0.063)			0.015 (0.010)
FSP × Community type			0.382** (0.133)			0.070** (0.022)
FSP × Hometown pressure			−0.018 (0.046)			−0.008 (0.007)
FSP × Neighbor composition			−0.228** (0.086)			−0.037* (0.014)
FSP × Perceived social status			0.068^†^ (0.036)			0.014* (0.006)
FSP × Withdrawal guarantee			−0.073 (0.084)			−0.015 (0.014)
Constant	−2.486^†^ (1.386)	−1.953 (1.356)	−1.501 (1.354)	−0.186 (0.642)	−0.151 (0.637)	−0.086 (0.636)
Vocation	In	In	In	In	In	In
Observations	15,997	15,997	15,997	15,997	15,997	15,997
Log likelihood	−7,674.357	−7,633.813	−7,615.807	−7,841.925	−7,803.646	−7,814.633
Akaike Inf. Crit.	15,420.710	15,341.620	15,325.610	15,763.850	15,689.290	15,731.270
Bayesian Inf. Crit.				16,070.960	16,004.080	16,122.800

Dependent variable: Local identity.

^†^*p* < 0.10;*p < 0.05; **p < 0.01; ***p < 0.001.

Standards errors are provided in parentheses.

### Descriptive analysis

Table 1 presented summary statistics for the final analytical sample. The descriptive statistics in Table 1 showed that 22.0% migrants who consider themselves the local. This result suggests a low overall level of migrants’ social integration, which sends an urgent signal to policymakers. Associated with the low level of social integration, the ISP and FSP of migrants kept low, 21.2 and 9.6%, respectively.

In terms of the migrants’ own characteristics, about 55.0% of the respondents were male, the mean age was 32.7 years old, and the vast majority (96.5%) was Han ethnicity. About 72.1% were newly married, and 25.4% were unmarried. The mean educational attainment was between junior high school and senior high school. 63.1% were the employee, and 86.0% were with the rural *hukou*. Particularly, the mean number of insurance migrants participating was low (1.1) in consideration of the constraints of *hukou* system. It indicates that migrants with rural *hukou* are brought with lots of disadvantages and restrictions for the welfare.

In terms of their work and income, they worked 8.8 h a day on average, and 44.2% signed a long-term contract with the employers. Their family income per month was 6,430.7 (SD = 7,051.5) RMB, while family expenditure was 3,139.1 (SD = 3,034.0) RMB, thus half of income was saved. The high save rated are consistent with Chinese concept of financial management.

When it comes to the community type, less than half of migrants lived in better communities (e.g., villa area and commercial housing community), ratio of local residents in their neighbors were relatively high, and they participated in the activities less than once (Mean = 0.7, SD = 1.0) on average.

Regarding immigration, most (94.9%) migrated for business, the mean duration of their immigration was 5.3 years, half of them (54.8%) were from a different province, and less than a third (29.6%) attended the local government-provided training. They had 3.79 (SD = 11.1) acres of cultivated land per capita.

### Main results

Hierarchical regression analysis and HLM were performed to examine our hypotheses. Table 2 presented the regression results of using the social identity as dependent variable.

The first three models (Model 1, Model 2, and Model 3) presented the regression coefficients which ignored the city level variables by using logit regressions. However, the other three models (Model 4, Model 5, and Model 6) were multilevel logit regressions, which included other city-level control variables and adopted the HLM.

Specifically, we adopted three steps to examine the interaction effect. We firstly added independent variables and other control variables, and the results were presented at Model 1 and Model 4, respectively. Then, we add the moderator in the basic model. As other moderators (community type, neighbor composition, hometown pressure, and withdrawal guarantee) were put in the first step model as controls, the second step only added perceived social status in the models (Model 2 and Model 4). Lastly, we added the interaction terms in the models (Model 3 and Model 6).

Measured as the ratio of between-cities variation to total variation, the intra-class correlation (ICC) was an indicator of using multilevel model. When ICC approaches 1, a multilevel is necessary (Goldstein, 2011). The ICC for null model in our research is 0.037. Although the ICC showed the differences between cities account for less than 5% variance, we followed the results of HLM to examine our hypothesis, and we listed the results of hierarchical regressions (first three models) as the comparison.

Consistent with prior research, we found migrants educational attainment (De Paola and Brunello, 2016), working pressure (Gallie, 2013), perceived social exclusion (Lu et al., 2013), household expenditure (Chen and Liu, 2016), community type (Li et al., 2012), neighbor composition (Li et al., 2012; Zou and Deng, 2020), community activities participation (Rublee and Shaw, 1991), migration years (Huang et al., 2018), and ranges of migration (Huang et al., 2018), and hometown pressure are all highly significant covariates, as shown in the Table 2. As seen in Model 4 and Model 5, the coefficients of ISP and FSP estimated by HLM were significant and positive, which confirmed support of H1a and H1b. Based on Model 5, the odds ratio of social identity for migrant who participated informal or formal organizations were more than double for who did not. Compared to the coefficients of ISP (*b* = 0.020, *t* = 2.476, *p* < 0.05), the coefficient of FSP (*b* = 0.031, *t* = 2.610, *p* < 0.001) are bigger, which implies that FSP plays a more important role than ISP in the social integration.

Model 6 presented results from interaction analysis. The significant and positive interaction terms of ISP × Community type (*b* = 0.037, *t* = 2.318, *p* < 0.05) and FSP × Community type (*b* = 0.070, *t* = 3.136, *p* < 0.01) confirm support H2a and H2b of the moderated effects of community type on the relationship between social participation and migrants’ social identity. The coefficient on ISP × Neighbor composition was 0.029 (*t* = 2.814, *p* < 0.01), and the coefficient on FSP × Neighbor composition is significant and negative at −0.037 (*t* = −2.567, *p* < 0.05). These findings suggest that when migrants’ neighbors are mainly composed by local residents, participating informal organizations will promote their social identity while participating formal organization impedes their social identity. Those results support our second group of hypotheses (H2a, H2b, H2c, and H2d).

The coefficients of hometown pressure were negative and significant in all six models, and the Model 6 reported the negative and significant coefficient for the interaction of ISP and hometown pressure (*b* = −0.014, *t* = −2.675, *p* < 0.01), thus supporting H3. We found that perceived social status positively predicted migrant’s social identity, and its coefficients ranges from 0.016 to 0.106 (*p* < 0.001). The Model 6 suggested that the moderating effects of perceived social status were positive and significant (*b* = 0.014, *t* = 2.502, *p* < 0.05), thus supporting H4.

In terms of the effects of withdrawal guarantee, the coefficients of it in not significant in the Model 4, Model 5, and Model 6. The interaction terms FSP × Withdrawal guarantee and ISP × Withdrawal guarantee in Model 6 were insignificant (*b* = −0.015, *t* = −1.067, *p* > 0.05; *b* = 0.015, *t* = 1.489, *p* > 0.05), thus not support H5a and H5b.

With respect to the effect of *hukou* system, the coefficients of *hukou* ranges from −0.059 (*p* < 0.001) to −0.341 (*p* < 0.001), at the same time, the coefficients of constraints of *hukou* ranges from −0.016 (*p* < 0.001) to −0.088 (*p* < 0.001). Consistent with the previous researches (Chan, 2009; Wei and Gao, 2017), there are adverse effects of *hukou* system on migrants’ social identity.

## Discussion and conclusion

Our research documented the coexistence of the positive impacts of FSP and ISP on migrants’ social identity and the different boundaries conditions for these two types of social participation.

Based on data from a 2014 nationwide survey and HLMs, this study investigated the social identity and willingness to integrate of 15,997 migrants across eight cities in China. Particularly, we examined the roles of FSP and ISP of migrants in destination cities in promoting their social identity. In addition, the moderating effects of community type, neighbor composition, hometown pressure, perceived social status and withdrawal guarantee were examined.

The effects of social participation on migrants’ social integration were demonstrated to be positive and significant. The results suggest that different social participation in host city can contribute to migrants’ psychological cognition and influence their identity. Different organizations serve as a platform for migrants to come into contact with the local culture and build social contacts. Through these platforms, the overall positive social identity of migrants can be enhanced by strengthening their sense of presence, higher familiarity of the local culture and the establishment of social relationships among local residents, thus improving their social integration. One interesting finding is that the effect of ISP on social identity is significant and positive, but the effect on willingness to integrate is not significant. The findings implies that migrants’ willingness for integration is changeable and that the positive effect of ISP on social integration may be covered by migrants’ negative immediate reaction caused by the adaptation process. Therefore, we need to recognize the critical role of ISP in the process of migrants’ social integration.

Importantly, the community type negatively moderates the effects of ISP and FSP on social integration. The results supported the previous studies by suggesting that the enhancement of the environment and atmosphere of the community is a crucial precondition for the adaption, assimilation and identification of the migrant population (Li et al., 2012; Zou and Deng, 2020). Meanwhile, the results imply the harmonious relationship and abundant social resources embedded in social networks can improve migrants’ positive perceptions for local residents, thus strengthening the positive effects of social participation on social integration. However, the neighbor composition has different moderating effects as migrants communicate and interact with local neighbors based on interests and hobbies, and this interaction pattern is more likely to be formed when there are more locals. This pattern is consistent with the characteristics of ISP which emphasizes the emotional resonance and the acceptance of individual differences.

We further found that migrants’ family background moderates the positive effect of ISP on social integration. Specifically, migrants’ hometown pressure leads to negative emotions and strong emotional needs, and the ISP provides a channel for them to relieve the pressure and acquire more emotional support. But when the ISP mitigates the negative emotions of migrants, the stress and pressure related to their hometown also come to their mind, which may impede their process of social integration. However, our findings revealed that withdrawal guarantee did not have a moderating effect between the relationship of ISP and social integration. This signifies that farmland is not an important source of survival for rural-urban migrants as most of them probably have other access to financial resources.

This study made an innovative contribution to the literature in two ways. First, this study focused on the different organizational structure characteristics between FSP and ISP, and examined the different roles that these two types of social participation play in the context of social integration. By applying social capital theory to the issue of social integration, this study enriched the literature on social integration and extended the research of social capital theory. Second, by using two dependent variables (i.e., social identity and willingness to integrate^3^), this study compared the difference between social identity and willingness to integrate, and captured the complexity of migrants’ psychological status.

Understanding the relationship between different kinds of social participation and social integration has important practical implications for China’s urbanization, reform of *hukou* system, and citizenship strategies. More attention should be given on positive roles of different organizations. Both FSP and ISP play different roles in different stages and aspects of social integration of migrants, which provides a good social foundation and multiple possible ways for the migrants. Therefore, progress can be achieved through including migrant population into the different organizations in the community and society according to their family background and other personal characteristics. In addition, considering that many migrants still live in poor communities with poor living conditions, more efforts should be made to improve migrants’ living conditions and increase their income, as well as encouraging social interactions between migrants and local residents. Furthermore, we call for a well-developed social service systems to facilitate migrants’ integration into their host communities by reducing the complexities and impracticalities of the *hukou* system. Further, the local government should consider reducing practices regarded as discriminatory for migrants.

## Limitation and further research

Despite important contributions to the literature, this research has some limitations that should be addressed in the future. First, it would be useful to examine the effects of social participation on other social integration, as social integration is a multi-level concept. In our research, we focused primarily on the perspective of the psychological level, however, the effects of social participation on cultural, economic, and political integration remain to be examined. Second, although we investigated the boundary effects of social participation on social integration by considering five important moderators, the mechanisms of social participation have not been unlocked in this study. Therefore, future research can examine the underlying mechanism of social participation in social integration. Third, the data we employed was cross-sectional, and future studies can use longitudinal data to reveal the impacts of social participation on social integration. Finally, this study mainly applied quantitative methods for conducting the research, future studies can consider adopting both qualitative and quantitative approach to examine how social participation influence social integration.

## Data availability statement

The original contributions presented in this study are included in the article/Supplementary material, further inquiries can be directed to the corresponding author.

## Author contributions

All authors listed have made a substantial, direct, and intellectual contribution to the work, and approved it for publication.

## Footnotes

1 The data can be download from the link: http://chinaldrk.org.cn/wjw/#/data/classify/population/topicList.

2 Because space is limited, we report the results in Supplementary material.

3 Please see the Supplementary material for details.
