# Supplementary material for: The effects of social participation on social integration
Source: Front Psychol. 2022 Sep 2;13:919592. doi: 10.3389/fpsyg.2022.919592 (PMC9479142; doi:10.3389/fpsyg.2022.919592)
Supplement: Supplementary file 1 [file Table_1.docx]

**Online Appendix**

To explore the different influencing factors of migrants’ social identity and their willingness to integrate, the regression results of willingness to integrate as the dependent variable are also reported below. The willingness to integrate reflects the subjective emotion and sentiments of migrants about the integration, while social identity mainly reflects the migrants’ psychological results of integration and is a stable value orientation state. The comparison of these two variables provides a perspective to interpret the perception of migrants. That is, we chose social identity as the main dependent variable, and whether our hypotheses are supported or not is based on the regression results using social identity as dependent variable.

To measure willingness to integrate of the migrants, we used a scale comprised of six items: (1) “I am willing to live with the locals in one block (community),” (2) “I am willing to be a colleague with local people,” (3) “I am willing to be a neighbor with the locals,” (4)“I am willing to make friends with local people,” (5) “I am willing to intermarry myself or my relatives with the locals,” and (6) “I am willing to become a member of the community.” Applying factor analysis, we extracted a single factor score to indicate the willingness to integrate. Specifically, we replaced the dependent variable with willingness to integrate, and used the HLM to examine the effects of social participation.

As shown in Table 1, Model 4 and Model 5 reported that the coefficients for formal organization participation are positive, with coefficients significantly different from zero at the 5% level. The results provided evidence of a positive relationship between formal organization participation and willingness to integrate. However, compared to the significant and positive effect of informal social participation on social identity, the positive effect of informal social participation on willingness to integrate is no longer significant. This finding implies that the willingness to integrate is a more changeable perception, and is more sensitive and susceptible to the immediate reactions, emotions, and impressions created in the communication and interaction between migrants and local residents in informal organization. Meanwhile, the immediate emotions and impression may be positive and negative depending on the contents and atmosphere, so the relationship between informal social participation and willingness to integrate is not significant.

Model 12 showed the significant interaction terms (*ISP* * *community type*, *ISP* * *Withdrawal guarantee*, *FSP* * *Withdrawal guarantee*). In other words, the moderating effects of community type and withdrawal guarantee between informal social participation and willingness to integrate were significant, and withdrawal guarantee moderates the effects of formal social participation on migrant’s willingness to integrate.

Overall, these results suggested the following: (1) formal social participation might be an important antecedent for migrants’ positive willingness to integrate; (2) the positive effect of informal social participation will be greater among migrants with better community type and sufficient withdrawal guarantee; (3) the positive effect of formal social participation will be greater among migrants with sufficient withdrawal guarantee.

| **Table 1**  Predictors of migrants’ willingness to integration by OLS and HLM.  Dependent variable: Willingness to integration | | | | | | |
| --- | --- | --- | --- | --- | --- | --- |
|  | *OLS* | | | *Linear mixed effects* | | |
|  | (1) | (2) | (3) | (4) | (5) | (6) |
| Informal Social participation (ISP) | 0.027 (0.061) | -0.001 (0.061) | -0.127 (0.082) | 0.076 (0.061) | 0.048 (0.061) | -0.055 (0.082) |
| Formal Social participation (FSP) | 0.235^**^ (0.088) | 0.233^**^ (0.088) | 0.271^*^ (0.116) | 0.188^*^ (0.088) | 0.185^*^ (0.088) | 0.195^†^ (0.117) |
| Community type | 0.088^†^ (0.052) | 0.103^*^ (0.052) | 0.046 (0.059) | 0.050 (0.052) | 0.063 (0.052) | 0.008 (0.059) |
| Neighbor composition | 0.252^***^ (0.032) | 0.235^***^ (0.032) | 0.202^***^ (0.037) | 0.167^***^ (0.034) | 0.150^***^ (0.034) | 0.117^**^ (0.038) |
| Hometown pressure | -0.046^**^ (0.017) | -0.035^*^ (0.017) | -0.019 (0.019) | -0.030^†^ (0.017) | -0.019 (0.017) | -0.002 (0.019) |
| Withdrawal guarantee | -0.035 (0.032) | -0.033 (0.032) | -0.092^**^ (0.036) | -0.044 (0.033) | -0.044 (0.033) | -0.107^**^ (0.037) |
| Perceived social status |  | 0.139^***^ (0.013) | 0.129^***^ (0.015) |  | 0.138^***^ (0.013) | 0.130^***^ (0.015) |
| ISP * Community type |  |  | 0.274^*^ (0.120) |  |  | 0.226^†^ (0.120) |
| ISP * Neighbor composition |  |  | 0.100 (0.077) |  |  | 0.103 (0.076) |
| ISP * Hometown pressure |  |  | -0.055 (0.039) |  |  | -0.063 (0.039) |
| ISP * Perceived social status |  |  | 0.052^†^ (0.031) |  |  | 0.048 (0.031) |
| ISP * Withdrawal guarantee |  |  | 0.223^**^ (0.075) |  |  | 0.235^**^ (0.075) |
| FSP * Community type |  |  | -0.030 (0.166) |  |  | 0.042 (0.167) |
| FSP * Hometown pressure |  |  | -0.054 (0.055) |  |  | -0.056 (0.055) |
| FSP * Neighbor composition |  |  | 0.101 (0.108) |  |  | 0.093 (0.108) |
| FSP * Perceived social status |  |  | -0.021 (0.043) |  |  | -0.026 (0.043) |
| FSP * Withdrawal guarantee |  |  | 0.190^†^ (0.104) |  |  | 0.200^†^ (0.103) |
| Constant | -1.963 (1.340) | -1.363 (1.337) | -1.031 (1.334) | -1.442 (2.960) | -1.142 (2.931) | -1.122 (2.935) |
| Controls | In | In | In | In | In | In |
| Observations | 15,997 | 15,997 | 15,997 | 15,997 | 15,997 | 15,997 |
| R^2^ | 0.222 | 0.227 | 0.228 |  |  |  |
| Adjusted R^2^ | 0.220 | 0.225 | 0.226 |  |  |  |
| Log Likelihood |  |  |  | -40,043.460 | -39,992.110 | -39,995.100 |
| Akaike Inf. Crit. |  |  |  | 80,166.920 | 80,066.220 | 80,092.200 |
| Bayesian Inf. Crit. |  |  |  | 80,474.030 | 80,381.010 | 80,483.730 |
| *Note:* ^†^ p < 0.1; ^*^ p<0.1; ^**^ p<0.05; ^***^ p<0.01; standards errors are provided in parentheses. | | | | | | |
